# Dr. William Baly

**Published:** 1861-05

**Authors:** 


DR. WILLIAM BALY, physician extraordinary to the queen, was suddenly killed by a railway accident on the 28th of January. He was fast rising in professional reputation, and was assistant physician and lecturer at St. Bartholomew's Hospital. A number of years ago he translated in an able manner Müller's "Elements of Physiology." He also contributed a number of valuable papers to the periodical press.

